# The complete chloroplast genome of *Sycopsis sinensis* Oliver

**DOI:** 10.1080/23802359.2020.1787900

**Published:** 2020-07-25

**Authors:** Ye Peng, Limei Yang, Jing Wei

**Affiliations:** Co-Innovation Center for Sustainable Forestry in Southern China, College of Biology and the Environment, Key Laboratory of State Forestry and Grassland Administration on Subtropical Forest Biodiversity Conservation, Nanjing Forestry University, Nanjing, China

**Keywords:** Chloroplast genome, *Sycopsis sinensis*, Hamamelidaceae, phylogenetic analysis

## Abstract

*Sycopsis sinensis* is a species of the genus *Sycopsis* Oliv in the Hamamelidaceae, and it is a native broadleaved evergreen woody plant species in China. Here, we sequenced, assembled, and analyzed the complete chloroplast (cp) genome of *S. sinensis*. The chloroplast genome of *S. sinensis* was 159,093 bp with 38.02% GC content, including a large single-copy (LSC) region of 87,841 bp, a small single-copy (SSC) region of 18,792 bp and two equal length inverted repeat (IR) regions of 26,230 bp. And, it contained 131 genes, including 37 tRNA genes, 8 rRNA genes, and 86 mRNA genes. Phylogenetic analysis strongly shows that S. sinensis has a close relationship with *Distylium macrophyllum*, whose posterior probability is 1.0.

*Sycopsis sinensis* Oliver, belonging to genus *Sycopsis* in Hamamelidaceae(Li et al., 2003), is a native broadleaved evergreen tree species distributed in Southen China. The largest population of *S. sinensis* as the dominant species was reported in the Houhe National Nature Reserve (Liu et al. 1999). Ago, domestic research on *S. sinensis* was limited to analysis of flora components and community level, but the research on *S. sinensis* from molecule level has not been reported so far. Here we firstly reported the complete chloroplast genomes of *S. sinensis* to promote further studies on population genetics, phylogeny and conservation biology in Hamamelidaceae.

Fresh leaves from one individual tree of *S. sinensis* were collected at the campus of Nanjing Forestry University (118.809603°E, 32.078520°N). The voucher specimen has been preserved at the Herbarium of Nanjing Forestry University (accession number Duan19121401). The whole genome was sequenced using Illumina NovaSeq highthroughput Sequencing platform based on Sequencing By Synthesis (SBS) technology. As a result, total 22,748,960 clean reads were produced and then used for the *de novo* assembly with NOVOplasty 2.7.2 (Dierckxsens et al. 2016). The annotated sequence has been submitted to GenBank with the accession number MT323104. Genome annotation, visualization, and tandem repeats identificationwas performed using the CpGAVAS pipeline (Liu et al. 2012).

The complete chloroplast genomes of *S. sinensis* was 159,093 bp. Like other species' chloroplast genome of Hamamelidaceae, the genome had a pair of IRs (26,230bp) regions separated by the LSC (87,841bp) and SSC (18,792bp) regions. The overall GC content was 38.02% for *S. sinensis*, whereas the GC content in the LSC, SSC and IR regions were36.19%, 32.47% and 43.06%, respectively. It contained 131 genes, including 37 tRNA genes, 8 rRNA genes, and 86 mRNA genes. Among those, 112 were unique and 19 (*ndhB, rpl2, rpl23, rps12, rps7, rrn16, rrn23, rrn4.5, rrn5, trnAUGC, trnI-CAU, trnI-GAU, trnL-CAA, trnN-GUU, trnR-ACG, trnV-GAC, ycf1, ycf15*, and *ycf2*) were duplicated in IR regions. Fourteen genes (eight protein-coding genes and six tRNA genes) contained one intron, and three genes (*clpP, rps12*, and *ycf3*) contained two introns.

In order to show position of *S. sinensis* in Hamamelidaceae, a phylogenetic tree was conducted by using MrBayes 3.2.6 (Ronquist et al. 2012) to infer. We selected and downloaded 16 species’ sequences of their complete chloroplast genomes from the National Center for Biotechnology Information. Sequences was aligned with MAFFT (Rozewicki et al. 2019) using '–auto' strategy and normal alignment mode across PhyloSuite1.1.16 (Zhang et al. 2020). ModelFinder (Kalyaanamoorthy et al. 2017) was used to select the best-fit model (GTR + F + I + G4) using AIC criterion. A Markov chain Monte Carlo (MCMC) was run for 1,000,000 generations with two parallel searches using four chains, each starting with a random tree. Trees were sampled every 100 generations. From the phylogenetic tree, we could discover that all species were clustered on the correct clades of family, *S. sinensis* and its congeneric species, *Distylium macrophyllum*, as sister group (posterior probability = 1.0; Figure 1).

**Figure 1.**
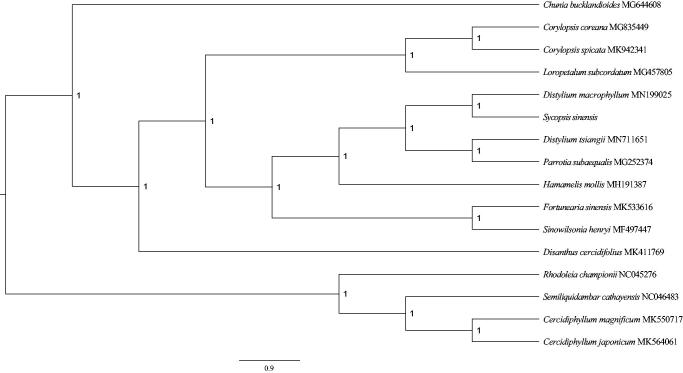
Phylogenetic relationships among 16 complete chloroplast genomes of Hamamelidaceae and Cercidiphyllaceae. Posterior probability values are given at the nodes. Cercidiphyllaceae are used as outgroups.
